# Characteristics and Seasonal Variations of Rhegmatogenous Retinal Detachment in the Eastern Black Sea Region of Turkey: 8-Year Results

**DOI:** 10.4274/tjo.galenos.2019.59140

**Published:** 2020-04-29

**Authors:** Hidayet Erdöl, Dilek Uzlu, Mehmet Kola

**Affiliations:** 1 Karadeniz Technical University Faculty of Medicine, Department of Ophthalmology, Trabzon, Turkey

**Keywords:** Rhegmatogenous retinal detachment, seasonal, cataract, surgery

## Abstract

**Objectives:**

To assess seasonal variations in the incidence of rhegmatogenous retinal detachment (RRD) in the Eastern Black Sea region of Turkey.

**Materials and Methods:**

Patients presenting due to primary RRD to a university hospital operating as a reference clinic in the region between 2011 and 2018 were evaluated retrospectively. Patients’ ages, sex, affected eye, and presentation times were recorded. Years were divided into months, quarters, seasons, and half-year periods, and these periods were analyzed in terms of differences in patient numbers.

**Results:**

Two hundred eighty-one eyes of 276 patients meeting the study criteria were included. The patients’ mean age was 60.2 years, and the male:female ratio was 1.35:1. Right and left eye rates were similar. Detachments were most common (49.4%) in the upper temporal quadrant. Eighty-nine patients (31.6%) had undergone uncomplicated phacoemulsification surgery a mean 2.7 years previously. The mean annual case number was 35.13±5.43, and no statistically significant variation was determined in case numbers by year (p=0.558). Analysis of all years revealed a monthly mean case number of 23.42±4.4, with the highest number of cases, 29 (10.3%), being seen in September and the lowest number, 13 (4.7%), in December. No statistically significant monthly variation was determined (p=0.613). Similarly, no statistically significant variation was observed in case numbers analyzed by quarter, season, or half-year (p>0.05).

**Conclusion:**

The incidence of cases of uncomplicated RRD does not exhibit seasonal variation in our region. We also think that since 31.6% had a history of cataract surgery, patients undergoing phacoemulsification surgery, even if uncomplicated, should be periodically assessed for detachment.

## Introduction

Rhegmatogenous retinal detachment (RRD) is one of the important vision-threatening diseases and requires an emergency approach. The annual incidence in the USA is 12/100,000, while studies from Asian and European countries have reported figures of 7-14/100,000.^1,2,3^ A study from China reported that the highest incidence was in the 40-59 age group, with an annual incidence of 14.4/100,000.^4^ An epidemiological study from New Zealand reported an incidence of RRD of 11.8 cases per 100,000, a mean age of 53.9 years, and a male:female ratio of 1.3:1.^5^

Studies investigating relations between RRD and seasonal changes have reported differing findings. One study from Lebanon reported a seasonal variation in the incidences of retinal detachment (RD), peaking in the spring and summer and at their lowest in fall and winter.^6^ Laatikainen et al.^7^ evaluated 301 patients in a university hospital in Finland over a 4-year period and also detected a significant increase in RD incidence in summer compared to colder winter months. In contrast, a study from Kuwait reported more RRD in winter.^8^ Another study also showed no seasonal variation in RRD.^9^

The purpose of the present study was to investigate seasonal variations and epidemiological characteristics of patients presenting with retinal detachment to a university hospital serving as a reference hospital in the Eastern Black Sea region over an 8-year period.

## Materials and Methods

Data for patients presenting with RRD to a tertiary hospital between January 2011 and December 2018 were evaluated retrospectively. The study complied with the principles of the Declaration of Helsinki and was approved by an ethics committee. Patients’ date of presentation, age, sex, affected eye, history of intraocular surgeries, bilaterality, and location and numbers of detachments were evaluated. Based on the patients’ date of presentation, numbers of patients were calculated in terms of months, quarters, seasons, and half-years. Seasons were defined as spring (March, April, May), summer (June, July, August), fall (September, October, November), and winter (December, January, February). Patients with previous uncomplicated cataract surgery were included in the study, and their surgery dates were recorded. Patients who developed detachment other than spontaneous RRD (traumatic or tractional), those with recurrent detachments, uveitis, glaucoma, aphakia, and history of ocular trauma or complicated cataract surgery, and those who underwent YAG laser capsulotomy as a result of posterior capsule opacification were excluded.

### Statistical Analysis

Statistical analysis was performed on SPSS Statistics 21.0 for Windows (SPSS, Chicago, IL) software. Numbers of patients by month, quarter, season, and half-year over the 8-year study period were determined, and the one-sample chi-square test was used to analyze the significance of differences among these time periods. P values <0.05 were considered significant.

## Results

Of 532 patients that presented with detachment, 281 eyes of the 276 patients who met the inclusion criteria were retrospectively analyzed. The patients’ mean age was 60.22±14.41 (23-88) years, 159 (57.6%) were men and 117 (42.5%) were women (ratio 1.35:1). One hundred forty-three (50.8%) right eyes and 138 (49.2%) left eyes were included, and the difference was not statistically significant (p=0.796). Bilateral detachment was present in 5 patients (1.8%), but there were no cases of simultaneous detachment. The interval between detachments in these 5 patients ranged between 5 and 53 months. Patients with detachment-related complaints or diagnoses for less than a month were included in the study. In addition, peripheral retinal degeneration was detected in the other eye in 34 patients (12.3%) and prophylactic 532 nm laser photocoagulation was applied. No RRD developed during follow-up in the patients receiving prophylaxis during the study period. Refractive values of the eyes with retinal detachment could not be obtained. However, eyes with axial length greater than 25 mm were excluded from the study.

Eighty-nine (31.6%) eyes were pseudophakic and had undergone uncomplicated cataract surgery a mean 2.7±2.8 years (2 months - 5.3 years) previously. Since there were no data regarding posterior vitreous detachment (PVD) before cataract surgery, the relationship between cataract surgery and PVD formation could not be evaluated in our study. Analysis of the retinal detachments revealed that 139 (49.4%) were in the superotemporal quadrant and 45 (16.0%) were in the inferotemporal quadrant. Retinal tears in multiple quadrants were observed in 69 eyes (24.5%).

The mean annual number of RRDs was 35.13±5.43 (29-44), and no significant differences were determined in patient numbers according to year (chi-square=5.595, p=0.558). Distributions of patients by year are shown in Table 1.

Analysis of monthly distributions over the 8-year study period revealed a mean monthly case number of 23.42±4.4, with the highest number of presentations occurring in September (n=29, 10.3%) and the lowest number in December (n=13, 4.7%). No statistically significant difference was determined between the months (chi-square=9.093, p=0.613) (Table 2).

Quarterly analysis (months 1-3, 4-6, 7-9, and 10-12) revealed that the highest number of cases, 79 eyes, occurred in the third quarter (28.1%), although no significant differences were determined between the quarters (chi-square=5.05, p=0.168) (Table 3).

In terms of seasons, the highest number of cases was seen in summer (n=77, 27.4%) and the fewest in winter (n=65, 23.1%). No statistically significant difference was observed (chi-square=1.121, p=0.772) (Table 3).

Analysis by half-year revealed that 147 (52.3%) cases were seen in the first 6 months and 134 (47.7%) in the second. The difference was not statistically significant (chi-square=0.601, p=0.438).

## Discussion

Retinal detachment is a severe, vision-threatening retinal disease requiring an emergency approach, and has an incidence of 7-14/100,000.^2,3^ Vision prognosis is linked to macular involvement.^10^ Even if anatomical success can be achieved following surgery in cases treated late, vision levels may still be low.

Several studies have described myopia as the most important risk factor for RRD.^11^ Another important risk factor is peripheral retinal degeneration, described as closely associated with myopia, and lattice degeneration has been reported in approximately 1 patient in 5.^12,13,14^ One study showed that lattice degeneration was present in 30% of RRD cases with atrophic holes.^15^ An epidemiological study from Taiwan investigated 2,359 patients and revealed a high prevalence of RRD between the ages of 50 and 69 and high myopia in 10.51% of cases. In addition, the condition was more common in men in all age groups.^16^ Similarly, another study from Western Australia showed that the risk of RD was greater in men and at more advanced ages.^17^ The mean age of the patients in our study was 60.22, and the male:female ratio was 1.35. We attribute the relatively higher mean age in our study to the lower incidence of high myopia in our region. However, our male:female ratio was similar to that in several previous studies.

Due to the high likelihood of a similar pathology in the other eye in patients with RRD, a detailed examination of the contralateral eye is essential.^18^ One study reported the incidence of lattice degeneration as 18.7% in the other eye, and the rates have been reported between 7 and 19% in various other studies.^2,12^ The rate of RD development in the fellow eye due to peripheral degeneration was reported as 24.5% in another study, and rates of bilateral RRD of 2.8 to 4.6% have been reported.^15,19^ Hajari et al.^20^ analyzed the risk of RRD in the fellow eye and reported figures of 1.3% per year, with a 5-year cumulative incidence of primary RRD in fellow eyes of 6.7±0.3%. Individuals with RRD in one eye have a 100-fold higher risk of RRD developing in the other eye, increasing still further with male sex and lens surgery, but declining with age. It was therefore concluded that patients require regular follow-up for at least 10 years.^20^ In our fellow eye analysis, we determined peripheral degeneration capable of causing RRD in 12.3% of cases, and these underwent laser prophylaxis. Bilateral RRD developed in 1.8% of cases during our study.

Another important risk factor for the development of RRD is a history of intraocular surgery, and RRD has been reported in 10 to 40% of cases following cataract surgery.^5,9,12,21^ Cataract surgery was shown to increase the risk of detachment at least 4-fold compared to the normal population, and there is a greater risk of cataract-related detachment in subsequent years, particularly among myopic subjects.^22^ However, since the follow-up periods in studies on this subject vary, there is also variation in the incidences reported. Post-cataract surgery RRD is reported to be more common in men compared to women.^22,24^ In one study from France, 2,680,167 cataract operations were performed in 2009 through 2012, and 62,065 detachment operations were carried out during the same period. The cumulative risk of RRD was 0.19% in non-operated patients and 0.99% in pseudophakic patients, with an odds ratio of 3.87.^25^ It has also been suggested that the incidence of RRD will rise still further due to the recent increase in cataract operations.^26^ In a prospective non-comparative series of 58 eyes without preoperative PVD at ultrasound examination, 58.7% developed PVD within the first year of phacoemulsification surgery, the majority occurring in the first postoperative month.^27^ A study from Spain retrospectively investigated 439 highly myopic eyes with a mean follow-up time of 61.5 months and determined an incidence of RRD of 2.7%. Patients were also assigned into two age-based groups at time of surgery. The incidence of RRD in patients aged 50 or less was 3.65%, compared to 2.52% in the over-50 group. Age at cataract surgery was correlated with risk of retinal detachment in high myopes.^22^ In a study from New Zealand, RRD developed in 33% of patients undergoing cataract surgery, and 50% of detachments post-cataract surgery occurred within 2 years of the cataract surgery and approximately 75% of these were within 12 months.^5^ In the present study, RRD developed after cataract surgery in 89 patients (31.6%) over the 8-year observation period, and detachment occurred at a mean 2.7±2.8 years (2 months - 5.3 years) after cataract surgery. This rate is consistent with the previous literature.

In terms of the tear site, Mitry et al.^12^ reported that 56% of tears occurred in the upper temporal quadrant, and that multiple tears were observed in 47.7% of cases. Chou et al.^15^ investigated 1,032 eyes from 1995 to 2001 and reported that 58.2% of tears were in the superior hemisphere and that multiple tears were present in 23.7% of patients. In our study, 139 (49.4%) of tears leading to detachment were in the superotemporal quadrant and 45 (16.0%) were in the inferotemporal quadrant. In addition, 24.5% (69 eyes) of patients had multiple tears. These data are also compatible with the previous literature.

Studies have also investigated the relation between RRD and seasonal variations and meteorological events in addition to known risk factors. In a study by Ivanisevic et al.^28^, 79 out of 272 cases between 1988 and 1999 occurred in summer, 71 in winter, 67 in spring, and 63 in fall. They observed no correlation between season and RRD. Li et al.^9^ also detected no seasonal variation in RRD case numbers in their study from Beijing. Mansour et al.^6^ retrospectively analyzed data pertaining to 211 consecutive patients over a 13-year period and reported 46 eyes with RRD in fall, 46 in winter, 62 in spring, and 57 in summer. Significant variation was observed, with RRD increasing in spring and summer (56%), when the weather is warmer, compared to winter and fall (44%) (p<0.05). They provisionally attributed this variation to exposure to sunlight and outdoor activities in the warmer weather.^6^ An 11-year (1999-2009) study of detachments across Taiwan revealed a significant seasonal relationship with the monthly incidence of RRD. The annual RD incidence rates were between 7.8 and 10.8 cases/100,000 during the study period, and the monthly RD incidence rates were positively associated with ambient temperature and negatively associated with atmospheric pressure.^29^ Laatikainenet et al.^7^ evaluated 301 patients over a 4-year period and reported more detachments in summer months than in winter. Paavola et al.^30^ reported a tendency for RRD to increase in spring and summer compared to winter. In contrast, another 7-year observation study from Kuwait reported more RRD in the winter months than in summer.^8^ In a study from Canada investigating whether there was any association between external environmental temperature and retinal detachment, only a relationship between tractional detachment and high temperature was detected; no correlation was observed between RRD and high temperature. The relationship between detachments and high temperature was reported to be more significant in individuals aged over 75.^31^ However, the majority of these studies of seasonal variations in RRD have been regional and involved different time intervals.

Although the incidence of RRD was higher in summer and fall in our study, no significant variation was observed compared to the other seasons. Due to the meteorological character of our region, exposure to sunlight is not high, and the number of sunny days is generally low. The difference in length between day and night in summer and winter is also not as great as it is in northern countries. The hypothesis that sunlight contributes to the development of RRD, as posited in some studies, can therefore be disregarded for our region.^6,7^ The most common month for RRD in our 8-year evaluation was September, at 10.3%, although this was not significantly different from the incidence in other months. The higher, albeit statistically insignificant, number of cases of RRD in September may be due to the greater engagement in agricultural and outdoor activities at that time as a result of the sociodemographic nature of the region. A significant and labor-intensive part of agricultural activities generally takes place in August and September. Therefore, we believe that these physically demanding activities may have contributed to the development of RRD. The incidence of RRD was relatively higher in the third quarter (28.1%) and in the summer (27.4%) compared to the other periods. Lower rates of RRD were observed in winter and December, although these were not statistically significant.

Our study involved an 8-year period, and since ours is the only reference center in the region, patients from our and surrounding provinces requiring vitreoretinal surgery are generally referred to our clinic. We regard this as sufficiently indicative of the numbers of RRD cases in our region. Ours is the first region-based research on the subject.

The principal limitations of this study are its single-center and population-based nature. Although our clinic is the only reference center in the Eastern Black Sea region, there is still a high probability of some patients presenting to centers outside it. An advanced database is therefore needed for all cases to be definitively evaluated. Increasing the sample size by evaluating all cases may thus elicit a more definitive result.

## Figures and Tables

**Table 1:**
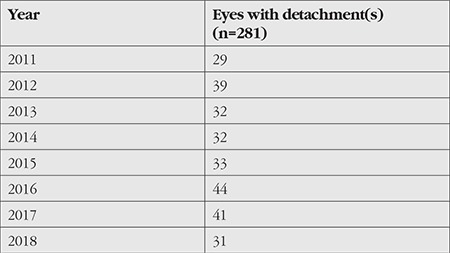
Annual distribution of patients presenting due to rhegmatogenous retinal detachment

**Table 2:**
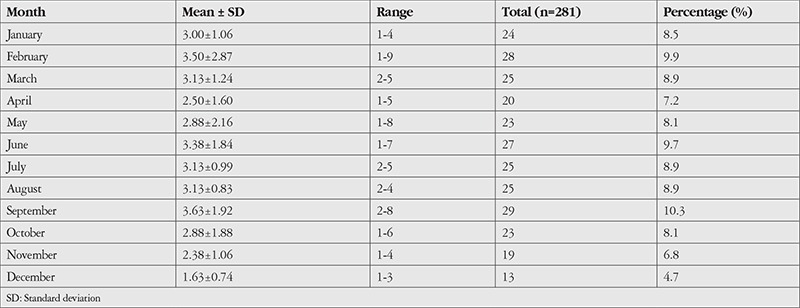
Mean and total monthly numbers and percentages of cases of rhegmatogenous retinal detachment over the 8-year study period

**Table 3:**
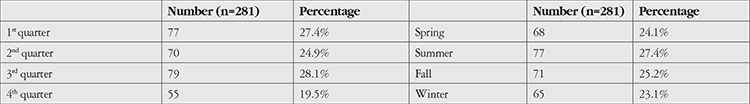
Distributions of cases of rhegmatogenous retinal detachment by quarter and season
